# An Unfortunate Controversy at Leeds

**Published:** 1916-10-28

**Authors:** 


### "An Unfortunate Controversy at Leeds."

With reference to the paragraph under the above heading in our issue of October 14, we have been requested to publish the following letter, written on October 5 by Mr. F. R. Spark, which appeared in the *Yorkshire Post.* We regret that the letter did not come to our knowledge until several days after our issue of October 14 was printed, as had we seen it we would certainly have given additional publicity to Mr. Spark's explanation:

THE LEEDS WORKPEOPLE'S HOSPITAL FUND.

To the Editor of the *Yorkshire Post.*

Sir,—Allow me to supplement the report contained in your issue of to-day of the meeting of the General Committee of the Leeds Workpeople's Hospital Fund by quite a brief statement, and beyond that I do not intend to take any further part in controversy.

Your report refers to a letter of May 19, 1914, addressed by officials of the Committee to Mr. Ramsden. That letter in terms explains to Mr. Ramsden the reasons why the Committee are unable to proceed with the erection and maintenance of a separate Home for children, and that the interest of the money had been used yearly in sending children to the Beckett's Convalescent Home for Children at Meanwood, and that they hoped to be able to provide a Children's Home in due course. Among the signatories to that letter, whose names you do not give, is Mr. Wormald, who makes the statement now reported on behalf of the Committee. Notwithstanding that letter, it is apparently contended that there never was any colour or suggestion of any arrangement or understanding that Mr. Ramsden's contribution should be used for a Children's Convalescent Home. That was the simple point of controversy between us.

It is quite true, as the Lord Mayor said, that he and Sir William Middlebrook, with Mr. E. O. Simpson, acting on my behalf, endeavoured to find "a half-way house," and it was upon the agreed suggestion of the Lord Mayor and Mr. Simpson that I wrote to the former, for communication to the Committee, a letter of which the following is a copy:—

> Dear Mr. Lupton,
>
> After further considering the points which were discussed by me with Sir William Middlebrook and yourself at our recent interview, I have come to the conclusion that the interests of the Leeds Workpeople's Hospital Fund, to which I have devoted so many years of my life, and in which I take such supreme interest, will not be furthered by a continuance of any discussion upon the points on which I have recently differed with the Workpeople's Hospital Fund Committee, and I therefore wish you to lay this letter before the meeting this evening, as being an expression of my intention to take no further controversial step in the matter.—Yours truly, Fred. R. Spark.

This letter, as I understand, was not laid before the meeting because it was not regarded by the Executive Committee as sufficient, but I stand by it, as it correctly expresses my final intention.

I equally adhere to my original letter to the General Committee, in which I alleged the purpose for which the £1,000 was contributed, though I was pressed by the Committee to withdraw that letter.—Yours, etc.,

October 5, 1916. Fred. R. Spark.

